# Metabolic Effects of Testosterone Replacement Therapy in Men with Functional Secondary Hypogonadism, Obesity and Type 2 Diabetes or Metabolic Syndrome: A Systematic Review

**DOI:** 10.3390/medsci14030418

**Published:** 2026-07-22

**Authors:** Christos Ntais, Apostolos Vantarakis, Ioanna P. Chatziprodromidou

**Affiliations:** 1 Epidemiology Program, School of Science and Technology, Hellenic Open University, 26335 Patras, Greece; avanta@upatras.gr (A.V.); ioannachatzi@med.upatras.gr (I.P.C.); 2 Department of Public Health, Medical School, University of Patras, 26504 Patras, Greece

**Keywords:** testosterone replacement therapy, hypogonadism, type 2 diabetes, metabolic syndrome, obesity, insulin resistance, systematic review

## Abstract

Background/Objectives: Functional secondary hypogonadism is frequent in men with obesity, metabolic syndrome, and type 2 diabetes mellitus (T2DM), but whether testosterone replacement therapy (TRT) produces clinically relevant metabolic benefits remains uncertain. This systematic review evaluated double-blind randomized placebo-controlled trials of TRT in obese men with functional secondary hypogonadism and either T2DM or metabolic syndrome. Methods: PubMed and Scopus were searched up to May 2026. Eligible studies enrolled adult men with biochemical hypogonadism and T2DM or metabolic syndrome, compared TRT with placebo, and reported metabolic outcomes. Open-label, single-blind, and non-randomized studies were excluded. Risk of bias was assessed with the Cochrane RoB 2 framework and certainty of evidence with GRADE. Results: Eight trials were included. The most consistent signal was improvement in body composition, mainly increased lean mass and reduced fat mass. Effects on insulin resistance were favorable in several trials but heterogeneous. GRADE certainty was low for fat mass, lean mass, and Homeostatic Model Assessment for Insulin Resistance (HOMA-IR), and it was very low for clamp-derived insulin sensitivity, HbA1c, fasting glucose, lipid outcomes, inflammatory and vascular surrogate markers, and long-term safety. Conclusions: Low-certainty evidence suggests that TRT may improve body composition and HOMA-IR in carefully selected men with functional secondary hypogonadism and metabolic disease, whereas effects on glycemic and lipid outcomes remain very uncertain. TRT should not be regarded as an antidiabetic or lipid-lowering intervention. Larger phenotype-specific trials with longer follow-up are needed.

## 1. Introduction

Obesity, metabolic syndrome, and type 2 diabetes mellitus (T2DM) are closely associated with low circulating testosterone in men [1,2,3,4,5]. The association is biologically plausible and clinically important: visceral adiposity, insulin resistance, chronic low-grade inflammation, and reduced sex hormone-binding globulin (SHBG) can lower total testosterone, while suppression of hypothalamic–pituitary–gonadal signaling may reduce free testosterone and produce a functional, potentially reversible, secondary hypogonadal state [1,5,6]. Conversely, testosterone deficiency may promote loss of lean muscle mass, accumulation of adipose tissue, and worsening insulin resistance, thereby establishing a bidirectional metabolic–hormonal cycle [7].

Functional secondary hypogonadism differs from classical primary testicular failure and organic hypothalamic–pituitary disease. In obese men with metabolic syndrome or T2DM, luteinizing hormone is often low or inappropriately normal, and testosterone may improve after weight loss or treatment of comorbidities [5,8]. Clinical guidelines therefore emphasize that testosterone replacement therapy (TRT) should be reserved for men with consistent symptoms and unequivocally low testosterone concentrations, after exclusion of reversible causes and contraindications [8]. The metabolic question is, when TRT is prescribed for genuine hypogonadism, whether it also improves glycemic control, insulin resistance, body composition, lipids, and inflammatory or vascular markers.

Previous reviews have often pooled heterogeneous populations, including men without pathological hypogonadism, men with primary hypogonadism, open-label interventions, observational cohorts, and diabetes-prevention trials [3,4,5]. Such inclusion may obscure whether TRT has metabolic effects in the specific phenotype of men with functional secondary hypogonadism associated with obesity and T2DM or metabolic syndrome. We therefore conducted a systematic review restricted to double-blind randomized placebo-controlled trials. The objective was to synthesize the metabolic effects of TRT in adult obese men with functional secondary hypogonadism and T2DM or metabolic syndrome. The review was deliberately conservative: trials were not included merely because they involved testosterone and diabetes, but only when the design and population were sufficiently close to the target phenotype. This approach prioritizes internal validity and clinical applicability over the number of included studies.

## 2. Materials and Methods

This review followed PRISMA 2020 reporting guidance [9]. PRISMA checklist is provided in Supplementary Table S1. A written internal protocol was prepared before screening and documented the review question and core methodological framework, including the search strategy, eligibility criteria, outcome domains, screening and data-extraction procedures, and the planned evidence synthesis. No substantive deviations from this framework occurred after screening began. The review protocol was not prospectively registered. PubMed and Scopus were searched up to May 2026 using the following search string: (testosterone OR testosterone replacement OR testosterone therapy OR testosterone replacement therapy OR TRT OR testosterone undecanoate OR testosterone cypionate OR testosterone enanthate OR testosterone propionate OR testosterone gel) AND (hypogonadism OR hypogonadotropic hypogonadism OR late-onset hypogonadism OR secondary hypogonadism OR functional hypogonadism OR testosterone deficiency) AND (type 2 diabetes OR diabetes mellitus type 2 OR metabolic syndrome OR obesity OR obese) AND (randomized OR randomised OR placebo OR double blind OR double-blind). PubMed was selected for its focused biomedical coverage and controlled indexing, whereas Scopus was selected for its broad multidisciplinary journal coverage and citation-tracking functionality. Previous comparative work has identified PubMed as particularly suitable for biomedical searching and Scopus as providing broad journal and citation coverage [10,11]. The electronic database search was supplemented by backward reference-list screening and forward citation searching of the included and other closely relevant publications.

Eligible studies were full-text, peer-reviewed, double-blind randomized placebo-controlled trials enrolling adult obese men with functional (non-organic) secondary hypogonadism associated with either T2DM or metabolic syndrome. Trials were required to compare any approved testosterone formulation with placebo and to report at least one metabolic outcome: HbA1c, fasting glucose, fasting insulin, Homeostatic Model Assessment for Insulin Resistance (HOMA-IR), hyperinsulinemic–euglycemic clamp-derived insulin sensitivity, lipids, body weight, body mass index (BMI), waist circumference, fat mass, lean mass, inflammatory markers, or vascular surrogate measures. Trials were excluded if they were open-label, single-blind, non-randomized, not placebo-controlled, involved men without pathological hypogonadism, did not specifically select for T2DM or metabolic syndrome, or represented secondary analyses of already included trials.

Data extracted from each eligible trial included country and setting, population, hypogonadism criteria, sample size, testosterone formulation, comparator, treatment duration, metabolic outcomes and key results. Feasibility of quantitative pooling was assessed separately for HbA1c, HOMA-IR and body-composition outcomes. Pooling was not performed because few trials contributed to each outcome and they differed in metabolic phenotype, testosterone formulation and route, treatment duration, baseline glycemic control, background antidiabetic therapy, outcome time points and summary metrics. HOMA-IR and clamp-derived measures were not treated as interchangeable, and body composition was assessed by non-equivalent methods, including anthropometry, DXA (Dual-energy X-ray Absorptiometry) and MRI (Magnetic Resonance Imaging). A qualitative synthesis was therefore performed by outcome domain. Because quantitative pooling was not considered clinically appropriate, certainty was assessed using Grading of Recommendations Assessment, Development and Evaluation (GRADE) within a structured outcome-specific narrative synthesis [12]. Judgments regarding inconsistency and imprecision were based on the direction, magnitude, and confidence intervals of the individual study estimates, total information size, and the extent to which clinically important benefit or harm could be excluded. Risk of bias was assessed using the Cochrane RoB 2 approach for randomized trials, considering randomization, deviations from intended interventions, missing outcome data, outcome measurement, and selective reporting [13]. Secondary reports derived from an already included trial were not treated as separate studies to prevent double-counting of participants and outcomes. These reports were consulted only when they provided supplementary methodological details, additional follow-up information, or clarification of prespecified metabolic endpoints relevant to the review.

Study eligibility was determined using a hierarchical decision process. Trial design was assessed first, with double-blind, placebo-controlled randomized trials considered essential for minimizing performance and detection bias. Eligible studies were then evaluated according to population characteristics to ensure that low testosterone reflected clinically relevant pathological hypogonadism rather than serving merely as an inclusion criterion in diabetes prevention or metabolic risk reduction trials. Finally, the underlying type of hypogonadism was considered. Studies predominantly involving men with classical primary hypogonadism, or mixed populations in which functional secondary hypogonadism could not be reliably isolated, were excluded from the main synthesis, even when relevant metabolic outcomes were reported.

**Figure 1:**
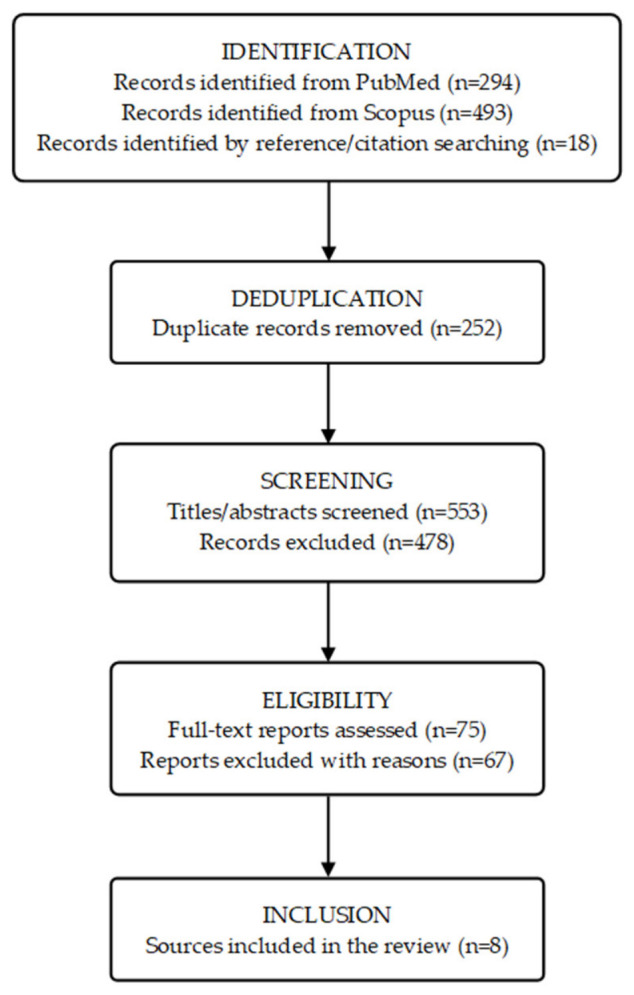
PRISMA 2020 flow chart of study selection.

The search and selection pathway is summarized in Figure 1.

## 3. Results

### 3.1. Study Selection Process

The database search identified 787 records (PubMed n = 294; Scopus n = 493), and 18 additional records were identified through reference/citation searching. After removal of 252 duplicates, 553 titles and abstracts were screened, of which 478 were excluded as irrelevant. A total of 75 full-text reports were assessed. Of these, 67 were excluded: 27 were non-randomized, open-label or single-blind; 23 did not specifically select for T2DM or metabolic syndrome; 13 did not require pathological hypogonadism; two were secondary analyses or sub-analyses; one addressed prevention/reversal of early T2DM rather than treatment of hypogonadism; and one included primary, secondary and mixed hypogonadism. Eight independent double-blind placebo-controlled trials were retained [14,15,16,17,18,19,20,21].

Important exclusions illustrate the boundaries of the evidence base. Kapoor et al. [22] was excluded despite being double-blind and clinically relevant because the population included primary, secondary and mixed hypogonadism, including Klinefelter syndrome. Gopal et al. [23] was excluded because the study population was not obese on average and did not represent the target metabolic phenotype. Heufelder et al. [24], Khripun et al. [25], and Janjgava et al. [26] did not meet the required double-blind placebo-controlled design. Konaka et al./EARTH [27] was not a double-blind placebo-controlled trial restricted to the target metabolic phenotype, and Shigehara et al./EARTH [28] was a secondary metabolic-syndrome analysis of that non-eligible parent trial. Wittert et al./T4DM [29] was excluded because it tested diabetes prevention or reversal in overweight men without pathological hypogonadism as a treatment indication. Further details are provided in Supplementary Table S2.

### 3.2. Characteristics of Included Studies

The included trials were published between 2010 and 2018 and included men with metabolic syndrome, T2DM, or both (Table 1). Testosterone undecanoate injections were used in five trials [14,15,17,18,21], testosterone gel in two [16,20], and testosterone cypionate in one [19]. Treatment duration ranged from 24 weeks to 12 months for the blinded comparison, with one trial reporting longer follow-up after unblinding [15]. Baseline glycemic control, background antidiabetic therapy, hypogonadism definitions, and achieved testosterone concentrations also varied across trials.

Two trials enrolled men with metabolic syndrome [14,15], whereas the TIMES2 trial included a mixed population of men with T2DM and/or metabolic syndrome [16]. The Moscow Study randomized 184 men with metabolic syndrome and hypogonadism to intramuscular testosterone undecanoate or placebo for 30 weeks; TRT reduced weight, BMI, waist circumference, insulin, leptin and HOMA-IR, and lowered CRP, IL-1β and TNF-α, without clear improvement in glucose or lipid profile [14]. Aversa et al. [15] studied 50 men with late-onset hypogonadism and metabolic syndrome; the placebo-controlled phase showed improved HOMA-IR, high-sensitivity C-reactive protein (hsCRP) and carotid intima-media thickness (CIMT), but the 24-month findings require caution because the blind was opened at 12 months and all participants subsequently received testosterone. TIMES2 randomized 220 hypogonadal men with T2DM and/or metabolic syndrome to testosterone gel or placebo. TRT reduced HOMA-IR by 15.2% at 6 months and 16.4% at 12 months, with HbA1c benefit emerging only in the T2DM subgroup at month 9 [16].

Five trials enrolled men with T2DM and functional secondary hypogonadism [17,18,19,20,21]. The BLAST trial reported improved HbA1c, particularly among men with baseline HbA1c above 7.5%, and reductions in waist circumference, weight and BMI in men without coexisting depression [17]. Gianatti et al. [18] found no improvement in HOMA-IR or HbA1c after 40 weeks of testosterone undecanoate, despite reduced fat mass and increased lean mass. Dhindsa et al. [19] specifically enrolled men with T2DM and functional secondary hypogonadism and used a hyperinsulinemic–euglycemic clamp; testosterone increased glucose infusion rate by 32%, increased lean mass, reduced subcutaneous fat, and lowered inflammatory mediators. Magnussen et al. [20] also used clamp methodology in men with T2DM on stable metformin monotherapy; testosterone improved lean and fat mass but did not improve HbA1c, peripheral insulin sensitivity, endogenous glucose production or substrate oxidation. Groti et al. [21] reported reductions in HbA1c, fasting glucose and HOMA-IR and improved flow-mediated dilation (FMD) after one year of testosterone undecanoate in hypogonadal men with T2DM.

### 3.3. Qualitative Synthesis by Metabolic and Safety Outcome

The most consistent signal was improvement in body composition (Table 1). Trials using DXA or MRI showed increased lean mass and reduced fat mass [18,19,20]. Changes in body weight and BMI may underestimate the effects of TRT on body composition, as increases in lean mass can partially or completely counterbalance reductions in fat mass. In Gianatti et al. [18] and Magnussen et al. [20], body composition improved without concomitant improvement in HbA1c or clamp-derived insulin sensitivity, highlighting that body recomposition is not necessarily equivalent to glycemic benefit. Reductions in waist circumference were reported in the Moscow Study [14], Aversa et al. [15] and BLAST [17].

Insulin resistance improved in several, but not all, trials. HOMA-IR decreased in the Moscow Study [14], Aversa et al. [15], TIMES2 [16] and Groti et al. [21]. Dhindsa et al. [19] provided direct physiological evidence by demonstrating improved clamp-derived insulin sensitivity, accompanied by increased adipose tissue expression of insulin-signaling genes and reductions in free fatty acids, CRP, IL-1β, TNF-α and leptin. However, Magnussen et al. [20] found no improvement in clamp-derived insulin sensitivity or endogenous glucose production under conditions of stable metformin monotherapy. This divergence suggests, but does not prove, that baseline adiposity, insulin resistance, diabetes duration, antidiabetic therapy, testosterone formulation, and the degree of testosterone replacement achieved during therapy may modify metabolic response.

HbA1c results were less consistent than insulin-resistance results. Groti et al. [21] and BLAST [17] reported improvement, particularly in men with poorer baseline glycemic control. TIMES2 showed HbA1c improvement at month 9 in the T2DM subgroup, but interpretation is complicated by medication changes permitted after 6 months [16]. In contrast, Gianatti et al. [18], Dhindsa et al. [19] and Magnussen et al. [20] reported no meaningful HbA1c improvement. The divergent findings may partly reflect treatment duration, baseline glycemic control and metabolic severity, but this interpretation is exploratory because no formal treatment-effect modifier analysis was possible.

Lipid findings were modest and variable. TIMES2 reported improvements in total cholesterol, LDL cholesterol and lipoprotein (a) in the metabolic syndrome subgroup, but HDL cholesterol decreased in all analysis groups [16]. BLAST reported lower total cholesterol [17], while Gianatti et al. [18] and Magnussen et al. [20] showed reductions in HDL cholesterol but no consistent total cholesterol, LDL, or triglyceride effect. Overall, no reproducible lipid-lowering effect was evident.

Inflammation and vascular surrogate markers were assessed less consistently. CRP and proinflammatory cytokines decreased in the Moscow Study [14] and Dhindsa et al. [19]. Aversa et al. [15] reported improved hsCRP and CIMT, and Groti et al. [21] reported improved FMD and CIMT. These findings are biologically plausible but remain exploratory surrogate outcomes. None of the included trials was designed to evaluate major adverse cardiovascular events or long-term safety.

Safety outcomes were inconsistently powered and reported. Most trials monitored hematocrit, hemoglobin, and prostate-specific antigen (PSA), and clinically significant increases in hematocrit were uncommon in the included trials. However, the sample sizes and durations were insufficient to evaluate major cardiovascular events, prostate cancer, or long-term thrombotic risk. The absence of observed harm in these small, short trials should not be interpreted as evidence of cardiovascular, prostate, or thrombotic safety. The present review therefore interprets safety descriptively and cannot establish long-term safety.

Table 2 summarizes the direction, GRADE certainty, and principal limitations across outcome domains.

### 3.4. Risk of Bias Assessment

Overall risk of bias was judged as “some concerns” for six trials and “high” for two [16,19]; no trial was judged to be at low risk overall. The most frequent limitations were incomplete reporting of allocation concealment, small samples, attrition, protocol deviations, last-observation-carried-forward or per-protocol analyses, multiple exploratory outcomes and open-label extensions. Outcome measurement bias was generally low because most outcomes were laboratory, anthropometric, DXA/MRI or clamp-derived. Detailed risk-of-bias judgments are provided in Supplementary Figures S1 and S2.

### 3.5. Certainty of Evidence (GRADE)

The GRADE assessment identified no high- or moderate-certainty outcomes (Table 2). Certainty was low for reductions in fat mass, increases in lean mass and changes in HOMA-IR. These ratings reflected serious risk-of-bias concerns across the contributing trials, together with inconsistency for fat mass and HOMA-IR or imprecision for lean mass. Certainty was very low for clamp-derived insulin sensitivity because the two small clamp trials were discordant and one had substantial differential attrition, for HbA1c, fasting glucose and lipid outcomes because effects were inconsistent and estimates were imprecise, and for inflammatory and vascular surrogate outcomes because of small samples, indirectness and inconsistent measurement. Certainty for long-term safety was also very low because the trials were short, events were rare, and none were designed to detect cardiovascular, prostate, or thrombotic harm. Detailed evidence profile and reasons for downgrading are provided in Supplementary Table S3.

## 4. Discussion

This systematic review suggests that TRT in men with functional secondary hypogonadism and metabolic disease may have selective metabolic effects. Its value lies not in establishing testosterone as a metabolic therapy, but in clarifying which outcomes may be affected when testosterone is replaced in men with confirmed hypogonadism. The most consistent signal was improved body composition, although the evidence base is small and affected by risk-of-bias concerns. Effects on insulin resistance were favorable in several trials but heterogeneous. Effects on HbA1c, fasting glucose and lipids were inconsistent and insufficient to support TRT as a metabolic drug independent of a hypogonadal indication. Formal GRADE assessment identified low certainty for fat mass, lean mass and HOMA-IR and very low certainty for clamp-derived insulin sensitivity, glycemic and lipid outcomes, inflammatory and vascular surrogate markers, and long-term safety; no outcome reached high or moderate certainty.

The heterogeneity is clinically informative but should not be overinterpreted. Trials with greater baseline metabolic burden, obesity or insulin resistance appeared more likely to show improvements in HOMA-IR or HbA1c, whereas well-controlled populations with relatively modest testosterone reductions and stable antidiabetic therapy sometimes showed neutral glycemic outcomes [18,20]. A secondary analysis of the Moscow Study found baseline HOMA-IR to be the most consistent predictor of HOMA-IR reduction after TRT [30]; however, these cross-trial and secondary observations are hypothesis-generating rather than definitive evidence of effect modification.

Several sources of clinical and methodological heterogeneity plausibly contributed to divergent findings: (1) formulation and route, because five trials used long-acting intramuscular testosterone undecanoate, two used transdermal gel and one used intramuscular testosterone cypionate; (2) blinded treatment duration, which ranged from 24 weeks to 12 months; (3) baseline glycemic control and metabolic burden; (4) severity and diagnostic definition of hypogonadism, including differences in total or free testosterone thresholds and achieved on-treatment concentrations; (5) background antidiabetic therapy, ranging from stable metformin to permitted medication changes; and (6) metabolic phenotype, with trials enrolling T2DM, metabolic syndrome or mixed populations. Outcome definitions and measurement methods also differed. Because these characteristics were unevenly distributed and often incompletely reported, reliable treatment-effect modification cannot be inferred.

Formal GRADE ratings reinforce the cautious interpretation. Low-certainty evidence means that the true effects on fat mass, lean mass and HOMA-IR may differ substantially from the estimates summarized here. Very low certainty for clamp-derived insulin sensitivity, HbA1c, fasting glucose, lipids, inflammatory and vascular surrogate markers, and safety means that the direction and magnitude of these effects remain highly uncertain. These ratings do not imply that benefit is absent; rather, further well-designed trials are likely to change the estimates and may alter the conclusions.

The review also emphasizes the importance of population selection. Functional secondary hypogonadism is often part of the metabolic disease process, but this does not mean that every obese man with low–normal testosterone is a candidate for TRT. Diagnosis requires repeated morning testosterone measurements, assessment of symptoms, consideration of SHBG and free testosterone when total testosterone is borderline or when SHBG-altering conditions are present, measurement of gonadotropins to distinguish primary from secondary hypogonadism, and exclusion of classical testicular or pituitary disease [31,32]. The included trials varied in how rigorously they applied these principles, which partly explains inconsistent metabolic results.

The discordance between improved body composition and neutral glycemic outcomes deserves particular attention. Testosterone commonly increases lean mass and reduces fat mass in hypogonadal men [33]; however, glycemic control depends on multiple factors, including beta-cell reserve, hepatic glucose production, skeletal-muscle insulin responsiveness and background antidiabetic therapy [34]. Consequently, HbA1c may remain unchanged despite favorable changes in body composition, particularly when diabetes is already well controlled or when visceral and hepatic fat are not substantially reduced [35]. This pattern was evident in Gianatti et al. [18] and Magnussen et al. [20]. Conversely, trials with higher baseline HbA1c or more severe insulin resistance, such as BLAST [17] and Groti et al. [21], were more likely to report glycemic benefit, although this comparison was not based on a formal subgroup analysis.

The inflammatory findings provide a biologically plausible mechanism but remain exploratory. Reductions in CRP, IL-1β, TNF-α, leptin and free fatty acids are mechanistically consistent with improved insulin signaling and endothelial function [36]. Nevertheless, these biomarkers were not measured consistently across trials and were not linked to hard clinical outcomes. Similarly, improvements in FMD or CIMT are hypothesis-generating rather than definitive evidence of reduced cardiovascular risk.

The review also emphasizes the importance of population and study design selection. Kapoor et al. [22] was excluded because it included primary, secondary, and mixed hypogonadism, including Klinefelter syndrome, despite favorable glycemic findings. Studies that lacked double-blind placebo control, including Heufelder et al. [24], Khripun et al. [25] and the EARTH study publications [27,28], were excluded because knowledge of allocation can increase performance and detection bias [37,38,39]. The T4DM study was excluded because it addressed diabetes prevention or reversal in overweight men without pathological hypogonadism, rather than treatment of functional secondary hypogonadism [29]. These exclusions may have reduced the apparent magnitude of benefit because some excluded studies reported favorable metabolic effects; however, including them would have reduced directness to the target population or increased design-related bias.

The findings have practical implications. For clinicians, the central message is that a metabolic comorbidity can be a reason to look for hypogonadism, not a reason to prescribe testosterone without establishing the diagnosis. Screening is most defensible in men with symptoms compatible with testosterone deficiency, sexual dysfunction, central obesity, T2DM or metabolic syndrome, especially when low testosterone is confirmed on repeat morning samples. Once treatment is started, metabolic changes should be interpreted as secondary benefits rather than primary treatment targets. HbA1c, fasting glucose and lipids should continue to be managed with established lifestyle and pharmacological interventions. Obese men with T2DM or metabolic syndrome should not receive testosterone solely to improve diabetes control. However, men with unequivocal functional hypogonadism may experience favorable changes in lean mass, fat mass, and, in some phenotypes, insulin resistance. Treatment decisions should integrate symptoms, repeated morning testosterone measurements, SHBG/free testosterone assessment when appropriate, LH/FSH evaluation, exclusion of organic pituitary or testicular disease, and careful monitoring of hematocrit, PSA, and cardiovascular risk [32].

This review has several limitations. First, only eight trials met the strict inclusion criteria, limiting confidence in subgroup interpretation. Second, the literature search was restricted to PubMed and Scopus. Although these databases were selected for their complementary biomedical and multidisciplinary citation coverage, and the search was supplemented by backward and forward citation searching, eligible studies indexed uniquely in Embase, Web of Science, the Cochrane Central Register of Controlled Trials (CENTRAL), or other information sources may have been missed. Evidence suggesting that sources beyond PubMed generally have only a modest impact on the results of systematic reviews of therapeutic interventions does not establish that a two-database search is exhaustive [10,11]. Third, although a written internal protocol was prepared before screening and no substantive deviations from its core methodological framework occurred during study selection or synthesis, it was not prospectively registered. Fourth, the trials differed in testosterone formulation, dose, achieved testosterone concentration, follow-up, baseline HbA1c, diabetes duration and background antidiabetic therapy. Fifth, several trials had small samples, attrition, or open-label extensions. Sixth, quantitative pooling was not performed after outcome-level feasibility assessment because the clinical and methodological differences could make pooled estimates misleading. Seventh, in the absence of pooled estimates and given the limited availability of outcome-level data, outcome-specific certainty of evidence was assessed narratively using the GRADE framework. Finally, most endpoints were intermediate metabolic or vascular markers, and the absence of observed harms cannot establish long-term cardiovascular, prostate or thrombotic safety.

Future trials should enroll men with clearly defined obesity-related functional secondary hypogonadism and T2DM or metabolic syndrome, use standardized diagnostic criteria, distinguish functional from organic hypogonadism and prespecify metabolic endpoints. Baseline stratification by HbA1c, HOMA-IR, waist circumference, SHBG, free testosterone and diabetes duration would help identify responders. Trials should also stabilize background antidiabetic and lipid-lowering therapy when metabolic efficacy is the main question, or otherwise prespecify how medication changes will be handled. Longer follow-up and adequate power are needed to test whether changes in insulin sensitivity and body composition translate into durable glycemic benefit or cardiovascular risk reduction.

## 5. Conclusions

In men with functional secondary hypogonadism associated with obesity, T2DM or metabolic syndrome, low-certainty evidence from eight heterogeneous double-blind placebo-controlled trials suggests that TRT may reduce fat mass, increase lean mass and improve HOMA-IR in some patients. Evidence for clamp-derived insulin sensitivity, HbA1c, fasting glucose, lipid effects and long-term safety has very low certainty. TRT should be viewed primarily as treatment for confirmed hypogonadism, with possible ancillary metabolic benefits, not as an antidiabetic or lipid-lowering intervention. The included trials do not establish cardiovascular, prostate or thrombotic safety. Rigorous long-term randomized trials are needed to identify responders and define long-term safety.

## Figures and Tables

**Table 1:** Characteristics and principal findings of included randomized placebo-controlled trials.

Study/Setting	Population and Phenotype	TRT Regimen	Comparator/Blinded Duration	Principal Metabolic Findings	Inflammatory/Vascular Surrogate Findings	Key Interpretive Caveat
Kalinchenko et al., 2010 (Moscow Study) [14] Russia; outpatient andrology/urology clinic	184 men, 35–70 years; metabolic syndrome and hypogonadism (TT < 12 nmol/L or cFT < 225 pmol/L)	Testosterone undecanoate 1000 mg IM at baseline and weeks 6 and 18	Matching placebo injection; 30 weeks	Weight, BMI and waist circumference decreased; the overall HOMA-IR group-by-time interaction was significant, but the week-30 and baseline-to-week-30 change comparisons were not significant; no clear fasting-glucose or lipid benefit.	CRP, IL-1β and TNF-α decreased	Short duration; multiple exploratory outcomes; overall RoB: some concerns
Aversa et al., 2010 [15] Italy; single center	50 men, 45–65 years; late-onset hypogonadism and metabolic syndrome	Testosterone undecanoate 1000 mg IM	Double-dummy placebo; 12-month blinded comparison, followed by open-label switch	HOMA-IR and fasting glucose improved; waist circumference decreased.	hsCRP and CIMT improved	Findings beyond 12 months are open-label; overall RoB: some concerns
Jones et al., 2011 (TIMES2) [16] 36 European outpatient centers	220 men, 37–88 years; hypogonadism with T2DM and/or metabolic syndrome	Dose-adjusted transdermal 2% testosterone gel	Placebo gel; 6-month stable-medication phase, 12 months total	HOMA-IR improved at 6 months; HbA1c was neutral at 6 months and improved only at month 9 in the T2DM subgroup; body-fat and lipid effects depended on analysis population.	No principal vascular endpoint; surrogate findings not consistently reported	Medication changes permitted after 6 months; attrition/LOCF; overall RoB: high
Hackett et al., 2014 (BLAST) [17] United Kingdom; seven primary-care centers	199 men, 33–83 years; T2DM and symptomatic testosterone deficiency	Testosterone undecanoate 1000 mg IM at weeks 0, 6 and 18	Matching placebo injection; 30 weeks	HbA1c was lower at weeks 6 and 18 but not significantly different at week 30 overall; HOMA-IR and fasting glucose were neutral; waist circumference improved at week 30.	No principal vascular or inflammatory endpoint	Positive HbA1c findings were transient or subgroup-specific; HOMA-IR was measured only in the severe subgroup; overall RoB: some concerns.
Gianatti et al., 2014 [18] Australia; tertiary center	88 men, 35–70 years; T2DM and TT ≤ 12 nmol/L; organic pituitary/testicular disease excluded	Testosterone undecanoate 1000 mg IM at weeks 0, 6, 18 and 30	Matching placebo injection; 40 weeks	No HOMA-IR or HbA1c improvement; fat mass decreased and lean mass increased; subcutaneous, but not visceral, fat decreased.	No principal vascular or inflammatory endpoint	Body-composition benefit without glycemic benefit; overall RoB: some concerns
Dhindsa et al., 2016 [19] United States; single center	44 men, 30–65 years; T2DM and hypogonadotropic hypogonadism	Testosterone cypionate 250 mg IM every 2 weeks, dose-adjusted	Saline placebo injection; 24 weeks	Clamp-derived glucose infusion rate increased by 32%; HOMA-IR improved; HbA1c was unchanged; lean mass increased and subcutaneous fat decreased.	CRP, IL-1β, TNF-α, leptin and other inflammatory mediators decreased	Small sample with differential dropout; overall RoB: high
Magnussen et al., 2016 [20] Denmark; university hospital	39 men, 50–70 years; T2DM on stable metformin and low bioavailable testosterone; organic hypogonadism excluded	Testosterone gel 50 or 100 mg/day	Placebo gel; 24 weeks	Lean and fat mass improved; no improvement in clamp-derived insulin sensitivity, HbA1c, endogenous glucose production or substrate oxidation.	No principal vascular or inflammatory endpoint	Small, relatively well-controlled metformin-treated population; overall RoB: some concerns
Groti et al., 2018 [21] Slovenia; diabetic outpatient clinic	55 men, 40–70 years; late-onset hypogonadism and T2DM treated with oral glucose-lowering therapy	Testosterone undecanoate 1000 mg IM every 10 weeks after loading	Placebo injection; 12 months	HbA1c, fasting plasma glucose and HOMA-IR decreased.	FMD and CIMT improved	Small single-center trial; surrogate vascular outcomes; overall RoB: some concerns

BMI, body mass index; cFT, calculated free testosterone; CIMT, carotid intima-media thickness; CRP, C-reactive protein; FMD, flow-mediated dilation; HbA1c, glycated hemoglobin; HOMA-IR, Homeostatic Model Assessment for Insulin Resistance; hsCRP, high-sensitivity C-reactive protein; IM, intramuscular; LOCF, last observation carried forward; RoB, risk of bias; T2DM, type 2 diabetes mellitus; TRT, testosterone replacement therapy; TT, total testosterone. Functional secondary hypogonadism is used as the umbrella term in the review; original trial labels are retained in study-specific population descriptions.

**Table 2:** Summary of findings and GRADE certainty across principal outcome domains.

Outcome Domain	Evidence Base	Direction of Effect	GRADE Certainty	Principal Limitations and Clinical Interpretation
Fat mass	5 RCTs; 445 randomized	Generally favorable. Four trials reported lower total or regional fat mass; TIMES2 was neutral in the intention-to-treat analysis, and visceral/hepatic fat often did not change.	Low	Downgraded for risk of bias and inconsistency across measurement methods and fat compartments. TRT may reduce fat mass, but the magnitude is uncertain.
Lean mass	4 RCTs; 225 randomized	Favorable. All four DXA-based trials reported increases in lean or fat-free mass (approximately 1.9–4.8 kg).	Low	Downgraded for risk of bias and imprecision because the evidence came from a small number of modest-sized trials without a pooled estimate.
HOMA-IR	8 RCTs; approximately 690 analyzed	Possible favorable effect. Approximately five trials reported improvement, whereas three were neutral or uncertain.	Low	Downgraded for risk of bias and inconsistency related to phenotype, background therapy, time point and analysis method.
Clamp-derived insulin sensitivity	2 RCTs; 73 analyzed	Uncertain. One trial reported higher glucose infusion rate, whereas the other found no improvement.	Very low	Downgraded for risk of bias, inconsistency and imprecision; the trials were small, used different clamp protocols and one had marked differential attrition.
Glycemic control (HbA1c and fasting glucose)	7 (HbA1c)/8 (fasting glucose) RCTs; more than 600 analyzed	Inconsistent. Clear final-time-point benefit was reported in two small trials; most other findings were neutral, transient or restricted to a subgroup/time point.	Very low	Downgraded for risk of bias, inconsistency and imprecision. Baseline control, medication changes and mixed T2DM/metabolic-syndrome populations complicate interpretation.
Lipid profile	8 RCTs; approximately 820 analyzed	No reproducible lipid-lowering effect. Some total/LDL reductions occurred, HDL decreased in several analyses, and triglycerides were generally neutral.	Very low	Downgraded for risk of bias, inconsistency and imprecision; background lipid-lowering treatment varied and no pooled effect was available.
Inflammatory markers	5 RCTs; approximately 330 analyzed	Possible favorable effect. CRP and selected cytokines decreased in some trials but were neutral in others.	Very low	Downgraded for risk of bias, inconsistency, indirectness and imprecision. Most biomarker analyses were secondary or exploratory and none was linked to clinical outcomes.
Vascular surrogate markers	2 RCTs; 105 randomized	Possible favorable effect. FMD and/or CIMT improved in both small trials.	Very low	Downgraded for risk of bias, indirectness and imprecision. These surrogate outcomes do not establish cardiovascular risk reduction.
Long-term safety	8 RCTs; 883 randomized	Indeterminate. Hematocrit and PSA increased in some trials, while major clinical events were rare and inconsistently reported.	Very low	Downgraded for risk of bias, serious indirectness and very serious imprecision. Blinded follow-up was only 24 weeks to 12 months and no trial was powered for major harm.

Participant counts are approximate randomized or analyzed denominators reported by the trials and varied by outcome and time point. Low certainty indicates limited confidence in the effect estimate; very low certainty indicates very limited confidence in the effect estimate.

## Data Availability

No new data were created or analyzed in this study. Data sharing is not applicable to this article.

## Supplementary Materials

The following supporting information can be downloaded at https://www.mdpi.com/article/10.3390/medsci14030418/s1; Table S1: PRISMA 2020 checklist; Table S2: Key full-text reports excluded after detailed assessment; Table S3: Detailed GRADE evidence profile and reasons for downgrading; Figure S1: Cochrane risk-of-bias summary by study and domain; Figure S2: Cochrane risk-of-bias bar graph by domain.

## Abbreviations

The following abbreviations are used in this manuscript.

BMI	Body mass index
cFT	Calculated free testosterone
CIMT	Carotid intima-media thickness
DXA	Dual-energy X-ray absorptiometry
EGP	Endogenous glucose production
FMD	Flow-mediated dilation
FPG	Fasting plasma glucose
GRADE	Grading of Recommendations Assessment, Development and Evaluation
GIR	Glucose infusion rate
HbA1c	Glycated hemoglobin
HOMA-IR	Homeostatic Model Assessment for Insulin Resistance
hsCRP	high-sensitivity C-reactive protein
IM	Intramuscular
MRI	Magnetic resonance imaging
PRISMA	Preferred Reporting Items for Systematic Reviews and Meta-Analyses
PSA	Prostate-specific antigen
SHBG	Sex hormone-binding globulin
T2DM	Type 2 diabetes mellitus
TRT	Testosterone replacement therapy
TT	Total testosterone
