# Supplementary material for: Metabolic Effects of Testosterone Replacement Therapy in Men with Functional Secondary Hypogonadism, Obesity and Type 2 Diabetes or Metabolic Syndrome: A Systematic Review
Source: Med Sci (Basel). 2026 Jul 22;14(3):418. doi: 10.3390/medsci14030418 (PMC13414107; doi:10.3390/medsci14030418)
Supplement: Supplementary file 1 [file medsci-14-00418-s001.zip › medsci-4412944-supplementary.pdf]

Supplementary Materials

Table S1. PRISMA 2020 checklist

PRISMA item	Topic	Location in manuscript
1	Title	Title page: title identifies the article as a systematic review.
2	Abstract	Abstract: structured summary of background, methods, results and conclusions.
3	Rationale	Introduction: rationale for focusing on functional secondary hypogonadism with obesity and T2DM or metabolic syndrome.
4	Objectives	Introduction: final paragraph states the objective.
5	Eligibility criteria	Materials and Methods.
6	Information sources	Materials and Methods: PubMed, Scopus and citation/reference searching up to May 2026.
7	Search strategy	Materials and Methods: complete search string reported.
8	Selection process	Materials and Methods and Figure 1.
9	Data collection process	Materials and Methods: extracted items listed.
10a	Data items: outcomes	Materials and Methods: metabolic outcomes specified.
10b	Data items: other variables	Materials and Methods and Table 1: population, intervention, comparator and duration.
11	Study risk of bias assessment	Materials and Methods; Supplementary Figures S1-S2.
12	Effect measures	Materials and Methods: study-specific effect estimates and directions were synthesized narratively; no pooled effect measure was calculated.
13a	Synthesis eligibility	Materials and Methods: all eligible RCTs entered qualitative synthesis.
13b	Data preparation	Materials and Methods: qualitative data extraction and grouping by outcome.
13c	Tabulation/visual display	Results: Figure 1, Tables 1-2 and Supplementary Tables S2-S3 and Figures S1-S2.
13d	Synthesis methods	Materials and Methods: outcome-level feasibility of pooling considered for HbA1c, HOMA-IR and body composition; no meta-analysis because of clinical and methodological heterogeneity.
13e	Heterogeneity exploration	Results and Discussion: heterogeneity structured by phenotype, formulation, route, duration, baseline glycemic control, hypogonadism severity, background therapy and outcome methods; see also Table 2.
13f	Sensitivity analyses	Results/Discussion and Supplementary Table S2: key near-eligible studies assessed qualitatively.
14	Reporting bias assessment	Materials and Methods/Discussion: formal assessment not performed because of small number and no meta-analysis.
15	Certainty assessment	Materials and Methods, Results, Discussion, Table 2 and Supplementary Table S3: GRADE assessment by outcome.

16a	Study selection results	Results and Figure 1.
16b	Excluded studies	Results, Figure 1, Discussion and Supplementary Table S2.
17	Study characteristics	Table 1.
18	Risk of bias in studies	Results and Supplementary Figures S1-S2.
19	Results of individual studies	Results and Table 1.
20a	Results of syntheses	Results and Table 2: structured narrative synthesis by outcome domain; detailed GRADE evidence profile in Supplementary Table S3.
20b	Statistical synthesis	Not applicable; no meta-analysis.
20c	Heterogeneity results	Results, Discussion and Table 2.
20d	Sensitivity analyses results	Discussion.
21	Reporting biases	Discussion.
22	Certainty of evidence	Results, Discussion, Table 2 and Supplementary Table S3.
23a	Summary of evidence	Discussion, Conclusions, Table 2 and Supplementary Table S3.
23b	Limitations of evidence	Discussion.
23c	Limitations of review process	Discussion.
23d	Implications	Discussion and Conclusions.
24a	Registration and protocol	Materials and Methods/Discussion: a written internal protocol was prepared before screening; it was not prospectively registered.
24b	Protocol access	Not publicly deposited.
24c	Protocol amendments	No substantive amendments to the review question, eligibility framework, outcome domains or planned narrative synthesis after screening began.
25	Support	Funding statement.
26	Competing interests	Conflicts of Interest statement.
27	Availability of data/materials	Data Availability Statement.

Table S2. Key full-text reports excluded after detailed assessment.

Report	Why it appeared potentially relevant	Precise reason for exclusion	Potential influence on interpretation if included
Kapoor et al., 2006 [22]	Double-blind placebo-controlled testosterone trial in men with T2DM and hypogonadism	Mixed primary, secondary and unspecified hypogonadal etiologies, including Klinefelter syndrome; the functional secondary subgroup could not be isolated	The favorable glycemic findings could increase the apparent benefit of TRT, but directness to obesity-related functional secondary hypogonadism would be reduced.
Gopal et al., 2010 [23]	Testosterone treatment in men with T2DM and hypogonadism	The cohort was not obese on average and did not represent the prespecified obesity/metabolic phenotype	Inclusion would broaden the population and reduce applicability to the review question; the likely influence on effect direction is uncertain.
Heufelder et al., 2009 [24]	Diet/exercise plus transdermal testosterone in newly diagnosed T2DM with subnormal testosterone	Did not meet the required double-blind placebo-controlled design; the lifestyle cointervention and knowledge of allocation increased susceptibility to performance bias	The reported favorable metabolic effects could make TRT appear more effective, but separation of testosterone effects from cointervention and expectancy effects would be difficult.
Khripun et al., 2019 [25]	Randomized testosterone study in newly diagnosed functional hypogonadism and T2DM	Did not meet the prespecified double-blind placebo-controlled design requirement	Favorable glycemic and endothelial findings would add supportive evidence, but with lower protection against performance and detection bias.
Janjgava et al., 2014 [26]	TRT study in men with T2DM and androgen deficiency	Did not meet the prespecified double-blind placebo-controlled design requirement	Inclusion could strengthen the apparent direction of metabolic benefit while reducing internal validity and comparability with the retained trials.
Konaka et al., 2016 (EARTH) [27]	Multicenter randomized trial in men with late-onset hypogonadism	Not a double-blind placebo-controlled trial restricted to men with T2DM or metabolic syndrome	Inclusion would broaden the phenotype beyond the review question and introduce greater design-related bias.
Shigehara et al., 2018 (EARTH subanalysis) [28]	Metabolic-syndrome subanalysis of the EARTH trial	Secondary analysis of a non-eligible parent trial; not an independent randomized comparison and inherits the parent trial's design limitations	Counting it as a separate trial would double-count participants and could overemphasize favorable subgroup findings.
Wittert et al., 2021 (T4DM) [29]	Large, 2-year, double-blind placebo-controlled testosterone trial in overweight/obese men enrolled in a lifestyle program	Tested prevention or reversal of early T2DM in men without pathological hypogonadism as a treatment indication; it addressed a different clinical question	Because of its size and favorable diabetes-prevention findings, inclusion could dominate the synthesis and incorrectly imply an antidiabetic indication for TRT in confirmed hypogonadism.
Tishova et al., 2024 [30]	Secondary report from the Moscow Study with additional predictor analyses and an open-label phase	Overlapping participants from an included trial; not an independent study and later follow-up included open-label treatment	Used only for contextual interpretation of potential response predictors; counting it separately would double-count participants and inflate apparent precision.

T2DM, type 2 diabetes mellitus; TRT, testosterone replacement therapy

Table S3. Detailed GRADE evidence profile and reasons for downgrading.

Randomized evidence started at high certainty. Because pooling was not appropriate, ratings were based on structured narrative synthesis of the contributing trials. Participant denominators varied by outcome and follow-up. Formal statistical assessment of small-study effects was not undertaken because no outcome was informed by at least 10 sufficiently comparable RCTs and no quantitative meta-analysis was performed. Publication bias was therefore considered qualitatively; no definitive signal was identified, but it could not be excluded.

Outcome	Studies / participants	Risk of bias	Inconsistency	Indirectness	Imprecision	Publication bias	Narrative effect summary	Certainty
Fat mass	5 RCTs; 445 randomized	Serious (-1) (a)	Serious (-1) (b)	Not serious	Not serious	Not strongly suspected; not downgraded; cannot be excluded	Four trials favorable; one large intention-to-treat analysis neutral; visceral/hepatic fat often unchanged.	**Low**
Lean mass	4 RCTs; 225 randomized	Serious (-1) (a)	Not serious	Not serious	Serious (-1) (c)	Not strongly suspected; not downgraded; cannot be excluded	All four DXA trials reported increased lean/fat-free mass (approximately 1.9-4.8 kg).	**Low**
HOMA-IR	8 RCTs; approximately 690 analyzed	Serious (-1) (a)	Serious (-1) (d)	Not serious	Not serious	Not strongly suspected; not downgraded; cannot be excluded	Approximately five trials favorable and three neutral/uncertain; estimates and time points were non-equivalent.	**Low**
Clamp-derived insulin sensitivity	2 RCTs; 73 analyzed	Serious (-1) (a,e)	Serious (-1) (e)	Not serious	Serious (-1) (e)	Not strongly suspected; not downgraded; cannot be excluded	Dhindsa et al. reported improved GIR; Magnussen et al. was neutral.	**Very low**
HbA1c	7 RCTs; approximately 610 analyzed	Serious (-1) (a)	Serious (-1) (f)	Not serious	Serious (-1) (f)	Not strongly suspected; not downgraded; cannot be excluded	Two small trials showed clear final-time-point benefit; other results were neutral, transient, subgroup-specific or potentially unfavorable.	**Very low**
Fasting plasma glucose	8 RCTs; approximately 820 analyzed	Serious (-1) (a)	Serious (-1) (g)	Not serious	Serious (-1) (g)	Not strongly suspected; not downgraded; cannot be excluded	Three trials favorable and five neutral; effect magnitude and clinical importance were uncertain.	**Very low**
Total and LDL cholesterol	8 RCTs; approximately 820 analyzed	Serious (-1) (a)	Serious (-1) (h)	Not serious	Serious (-1) (h)	Not strongly suspected; not downgraded; cannot be excluded	Some reductions in selected trials/subgroups, but most findings were neutral and no reproducible lipid-lowering effect emerged.	**Very low**
HDL cholesterol	8 RCTs; approximately 820 analyzed	Serious (-1) (a)	Serious (-1) (h)	Not serious	Serious (-1) (h)	Not strongly suspected; not downgraded; cannot be excluded	HDL decreased in several analyses and was neutral in others; clinical implications were uncertain.	**Very low**
Inflammatory markers	5 RCTs; approximately 330 analyzed	Serious (-1) (a)	Serious (-1) (i)	Serious (-1) (i)	Serious (-1) (i)	Not strongly suspected; not downgraded; cannot be excluded	CRP and selected cytokines improved in some trials but not others; most biomarker analyses were secondary or exploratory and none was linked to clinical outcomes.	**Very low**
Vascular surrogate markers	2 RCTs; 105 randomized	Serious (-1) (a,j)	Not serious	Serious (-1) (j)	Serious (-1) (j)	Not strongly suspected; not downgraded; cannot be excluded	FMD and/or CIMT improved in two small trials, but these were surrogate outcomes.	**Very low**
Long-term safety	8 RCTs; 883 randomized	Serious (-1) (a)	Not serious	Serious (-1) (k)	Very serious (-2) (k)	Not strongly suspected; not downgraded; cannot be excluded	Events were rare; follow-up was short; trials were not designed or powered to detect long-term harm.	**Very low**

(a) No included trial was at low risk of bias overall; six had some concerns and two were at high risk.
(b) One large intention-to-treat analysis was neutral, and reductions in total/subcutaneous fat were not consistently accompanied by lower visceral or hepatic fat.
(c) Only four modest-sized trials contributed, and no pooled confidence interval was available.
(d) HOMA-IR findings varied across metabolic phenotype, background treatment, follow-up, analysis population and summary metric.
(e) The two clamp trials were small and discordant; Dhindsa et al. [19] had substantial differential attrition, whereas Magnussen et al. [20] used a stable-metformin population and a different clamp protocol.
(f) HbA1c findings ranged from benefit to no benefit or possible worsening; some positive findings were transient, subgroup-specific or occurred after medication changes were permitted.
(g) Fasting-glucose effects were favorable in only a minority of trials and were often estimated imprecisely.
(h) Total, LDL, HDL and triglyceride effects differed by trial and background lipid-lowering therapy; no pooled estimate was available.
(i) Inflammatory biomarkers were exploratory, measured with non-uniform panels and not linked to patient-important clinical outcomes.
(j) FMD and CIMT are surrogate outcomes; only two small trials contributed and one had baseline imbalance in CIMT.
(k) Blinded follow-up ranged from 24 weeks to 12 months, major events were rare, and the evidence is indirect and severely underpowered for long-term safety.
Certainty categories: low certainty = limited confidence in the estimate; very low certainty = very limited confidence. No outcome reached high or moderate certainty.

Figure S1. Cochrane risk-of-bias summary by study and domain.

Study	D1	D2	D3	D4	D5	Overall
Kalinchenko et al. 2010	yellow	green	green	green	yellow	yellow
Aversa et al. 2010	yellow	yellow	yellow	green	yellow	yellow
Jones et al. 2011/TIMES2	yellow	yellow	red	green	yellow	red
Hackett et al. 2014/BLAST	green	green	green	green	yellow	yellow
Gianatti et al. 2014	green	green	yellow	green	yellow	yellow
Dhindsa et al. 2016	yellow	green	red	green	yellow	red
Magnussen et al. 2016	green	green	yellow	green	yellow	yellow
Groti et al. 2018	yellow	green	yellow	yellow	yellow	yellow

D1: bias arising from the randomization process,
D2: bias due to deviations from intended intervention,
D3: bias due to missing outcome data,
D4: bias in measurement of the outcome,
D5: bias in selection of the reported result
green = low risk, yellow = some concerns, red = high risk

Figure S2. Cochrane risk-of-bias bar graph by domain

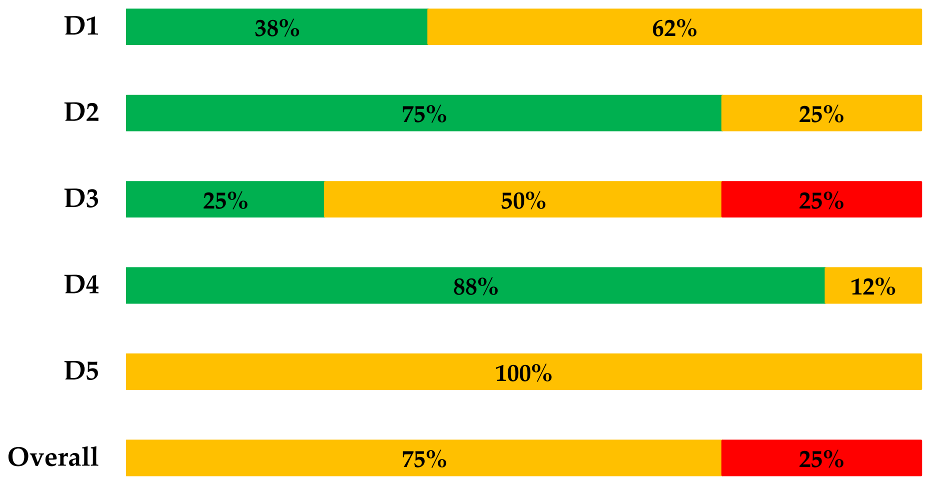

D1: bias arising from the randomization process,
D2: bias due to deviations from intended intervention,
D3: bias due to missing outcome data,
D4: bias in measurement of the outcome,
D5: bias in selection of the reported result
green = low risk, yellow = some concerns, red = high risk

Notes: The overall judgment was "some concerns" for six trials and "high risk" for two trials. Jones et al. [16] was rated "high risk" overall because attrition, protocol deviations and last-observation-carried-forward methods affected the 12-month interpretation; the initial 6-month stable-medication phase was more robust. Dhindsa et al. [19] was rated "high risk" overall because dropout was markedly higher in the placebo arm. Aversa et al. [15] was considered interpretable for the 12-month blinded comparison, whereas the 24-month results were considered open-label extension data after unblinding.
